# Functional Outcome Changes in Surgery for Pituitary Adenomas After Intraoperative Occurrence of the Trigeminocardiac Reflex: First Description in a Retrospective Observational Study

**DOI:** 10.1097/MD.0000000000001463

**Published:** 2015-09-18

**Authors:** T. Chowdhury, C. Nöthen, A. Filis, N. Sandu, M. Buchfelder, Bernhard Schaller

**Affiliations:** From the Department of Anesthesia and Perioperative Medicine, University of Manitoba, Winnipeg, Canada (CT); Department of Neurosurgery, University of Erlangen-Nuremberg, Germany (NC, FA, BM), and Department of Research, University of Southampton, Southampton, UK (SN, SB).

## Abstract

Trigeminocardiac reflex (TCR) represents now a nearly ubiquitary phenomenon in skull base surgery. Functional relevance of the intrainterventional TCR occurrence is hitherto only proven for vestibular schwannoma.

In a retrospective observational study, 19 out of 338 (8%) enrolled adult patients demonstrated a TCR during transsphenoidal/transcranial surgery for pituitary adenomas. The 2 subgroups (TCR vs non-TCR) had similar patient's characteristics, risk factors, and histology. Preoperatively, there was a similar distribution of normal pituitary function in the TCR and non-TCR subgroups. In this TCR subgroup, there was a significant decrease of that normal pituitary function after operation (37%) compared to the non-TCR group (60%) (*P* < 0.03). The TCR subgroup therefore demonstrated a 3.15 times (95%CI 1.15–8.68) higher risk for non-normalizing of postoperative pituitary function compared with the non-TCR subgroup (*P* < 0.03).

It is presented, for the first time, an impact of TCR on the functional hormonal outcome after pituitary surgery and strongly underline again the importance of the TCR in clinical daily practice. As a consequence, TCR should be considered as a negative prognostic factor of hormonal normalization after surgery for pituitary adenomas that should be included into routine practice.

## INTRODUCTION

Trigeminocardiac reflex (TCR) is a well-established brainstem reflex elicited by the mechanical/thermical stimulation of any sensory branch of trigeminal nerve along its pathway.^1–13^ It commonly manifests as arterial hypotension, bradycardia/asystole, apnoea, and gastric hypermotility.^3,4,13–23^ TCR is usually defined as a simultaneous fall in mean arterial blood pressure (MABP) and heart rate (HR) by at least 20% of the base line values.^3^ After its first description in human by Schaller et al in 1999,^3,13,20,21,23^ numerous investigators have focused on the pre- and intraoperative risk factors associated with intraprocedural TCR occurrence (see for example).^10–11,21–22,24–25^ Although much experimental and clinical works have been already published on the pathogenesis of this reflex during the last few years (e.g.,^26^); many important aspects still remain unanswered. Until date, different neurosurgical procedures have been associated with the occurrence of TCR^1–3,5,7,12,26–38^ but only in vestibular schwannoma resection, the occurrence of TCR could actually linked with functional (hearing/vestibular) outcome.^7,10,39–40^ The association of this reflex with the worse functional outcome in other types of surgeries/interventions remain yet to be further determined.

Pituitary surgery is a common but also demanding skull base procedure^41–44^ and has also often been linked with occurrence of intraoperative TCR episodes.^5,8,12,27–28,45–67^ In another context, it could been shown that the gender has an important influence on pituitary adenomas; especially in frequency and in disease patterns.^26,32–34,36,39,46^ But for the TCR nothing is known about a gender-dependence in pituitary adenoma function. However, the effects of such adverse events as induced by the TCR on postoperative pituitary function have never been explored as well. On the basis of above-mentioned factors and the previous observation in pituitary surgery, therefore, this retrospective observational study aims to elucidate the impact of intraoperative occurrence of TCR on the postoperative pituitary hormonal functions special reference is given to the gender differences.

## MATERIAL AND METHODS

This is a retrospective observational study, including all consecutive patients who underwent elective pituitary surgery via transsphenoidal and/or transcranial approach, in the Department of Neurosurgery in the University Hospital of Göttingen in Germany during the time period of 2002 to 2005. Local institutional review board approval was obtained as a part of the dissertation project.

### Inclusion and Exclusion Criteria

All adult patients (more than or equal to 18 years of age) with a definitive histological diagnosis of pituitary adenomas were included into the study. So that a total of consecutive 345 patients of all adult age groups of either gender were included in this study. Four patients of this cohort (1%) with significant medical comorbidities including uncontrolled arterial hypertension, uncontrolled diabetes mellitus, and pre-existing cardiac disease (HR < 50/min, arrhythmias, recent myocardial ischemia [past 6 month]) were excluded from the study. Further 3 patients of this cohort (1%) were also excluded because of substantial missing data/records, so that finally 338 patients could be included.

The medical records of all included patients were systematically reviewed and analyzed statistically.

### Preoperative Examinations

Before operation, all 338 patients had underwent detailed clinical and laboratory examination as well as neuroradiological examinations (magnetic resonance imaging). In addition, detailed preanaesthetic evaluations were done.

### Anaesthetic Technique

The surgical procedure was performed under a standardized anesthesia protocol as described earlier.^8,27–28,41–43^ All the patients were kept fasting for at least 6 hours before the surgery and were premedicated with oral midazolam. Routine intraoperative monitoring included electrocardiography, measurement of end-tidal concentration of carbon dioxide and isoflurane, pulse oximetry, and oesophageal temperature. Only in cases where a craniotomy was performed an indwelling radial artery catheter was inserted for continuous monitoring of blood pressure and for the intermittent blood gas analysis. HR and oxygen saturation were monitored continuously whereas blood pressure in 5 minutes intervals in cases of transsphenoidal surgery following the standard anesthesiology procedures in the hospital. General anesthesia was induced with propofol (2–3 mg/kg), sufentanyl (0.25 mcg/kg), and rocuronium (0.6 mg/kg). Anesthesia was maintained with desflurane (1–1.5 minimum alveolar care); additional boluses of sufentanil and rocuronium were administered if necessary.

### Surgical Procedure

Standard microsurgical procedures including transsphenoidal as well as transcranial procedures described earlier^41,42^ were performed by a senior, very experienced neurosurgeon. Following the procedure, all patients were transported to the intermediate care unit for further observation.

#### Defining TCR

Any simultaneous drop of 20 % or more, from the baseline in MABP and mean HR during tumor manipulation, was defined as TCR as defined in the initial work of Schaller et al and is now generally accepted.^3^ The occurrence must be preceded with definitive stimuli including physical, chemical, or electrical manipulation at or near the vicinity of trigeminal nerve (peripheral or the central part).^3^ Additionally, a clear cause-effect relationship is required.^3^ According to this strictly defined occurrence of the TCR, these patients were divided into 2 principal groups (TCR vs non-TCR) for further statistical analysis.

#### Data Collection of Outcome Variables and Pituitary Function

Outcome variables on demographic data (gender, age, and BMI), type of surgery (transsphenoidal/transcranial), histological type (WHO classification), and tumor size (see below for details)/diameter of pituitary adenoma (Hardy classification; see^5,6,8^ for details), preoperative hormone levels (see below for details), comorbidities (see above for details), medication administered, routine blood work (investigations), intraoperative hemodynamic including HR, arterial blood pressure, and postoperative hormonal status (see below for details) were documented.

As per the definition macroadenomas exhibited a tumor diameter of >1 cm, whereas microadenomas were ≤1 cm.^41–43^ The tumor size was measured as largest tumor diameter on magnetic resonance imaging.^8^

In cases, where the MABP was recorded directly, it was taken as such, otherwise it was calculated from the systolic and diastolic blood pressure. The recorded concomitant HR was also used as a baseline. Then, after the start of the operation, the lowest recorded MABP, and HR were taken. The percent reduction in MABP and HR was then calculated from both measurements.

Patients were further divided into 3 subgroups preoperatively according to their hormonal status: those with “normal” pituitary function, “decreased secretion,” or “increased secretion” of pituitary hormones.^43^ For that classification we have used the following definitions prolactinoma—normalization of postoperative serum prolactin levels at 7 days and 3 months. Cushing disease—normalization of free cortisol in 24-hour urine, dexamethasone suppression test (2 mg at night) with morning serum cortisol <1 mg/L are considered to be in remission acromegaly—normalization of basal growth hormone levels and insulin-like growth factor-1 suppression of growth hormone <1 mcg/L in 60 minutes after loading with 100 g glucose.

### Statistical Analysis

All the statistical analyses were performed using statistical software JMP/SAS Institute Inc. (USA) on a commercially available computer. Data are presented as number (percentage). Data were tested for normality using the D’Agostina & Person omnibus normality test. Data normally distributed are represented by mean (standard deviation [SD]) and numbers (percentage). For categorical, independent outcome variables, the Chi-square test were used while the continuous, independent variables were compared using the 2 sample *t*-test or the Mann–Whitney test. Because the overall number of TCR was small, the Yates correction factor was used to calculate Chi-square statistics. We also calculated relative risk and 95% confidence interval.

The alpha-level of significance was set at *P* < 0.05. With the number of patients available for review our study had a power of >80% to discern difference between the various groups.

## RESULTS

In the present retrospective observational study, 338 consecutive patients who underwent a transsphenoidal (n = 337) or transcranial (n = 1) operation for pituitary adenoma resection were enrolled. In 19 patients (6%) TCR was observed, and these patients were categorized as TCR subgroup (see Table 1). The remaining 319 patients (94%), in whom no TCR was elicited during transsphenoidal/transcranial operation for pituitary adenoma comprised of the non-TCR subgroup. There was no statistically significant difference between the TCR and non-TCR groups in terms of gender, age, tumor diameter, BMI, type of surgery, and preoperative pituitary hormonal status (see Table 2). The tumor histology showed no significant difference between TCR and non-TCR (see Table 3). During the operation, anticholinergic drugs were administered in 10 patients (52.6%) of the TCR group and in 19 patients (6.0%) of the non-TCR group for treatment/prevention of bradycardia.

**TABLE 1:**
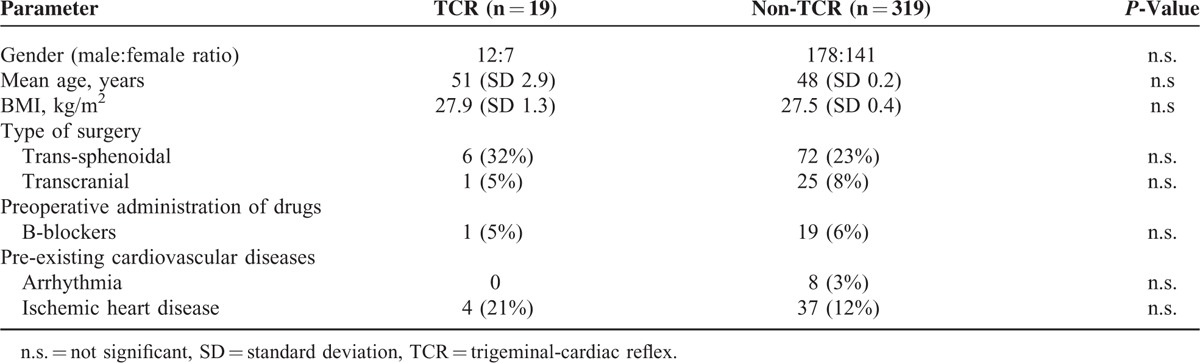
Demographic Profile of the 2 Subgroups

**TABLE 2:**
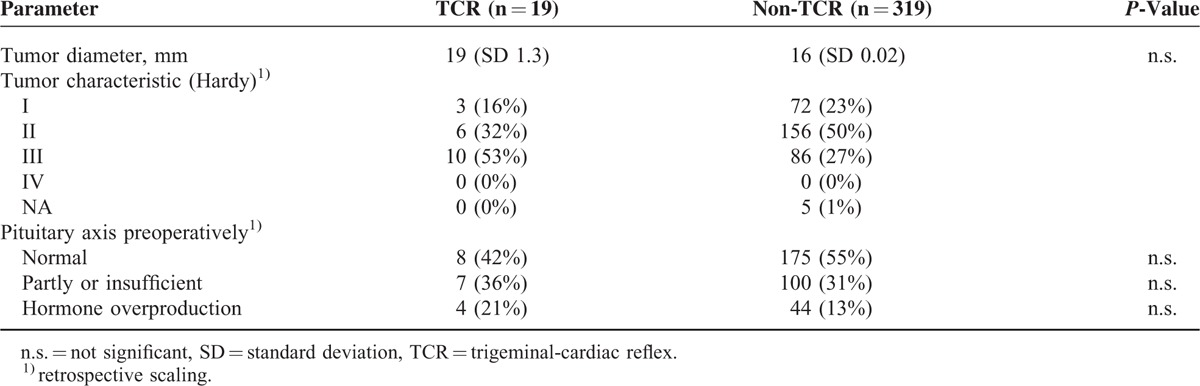
Pituitary Adenoma Characteristics of the 2 Subgroups

**TABLE 3:**
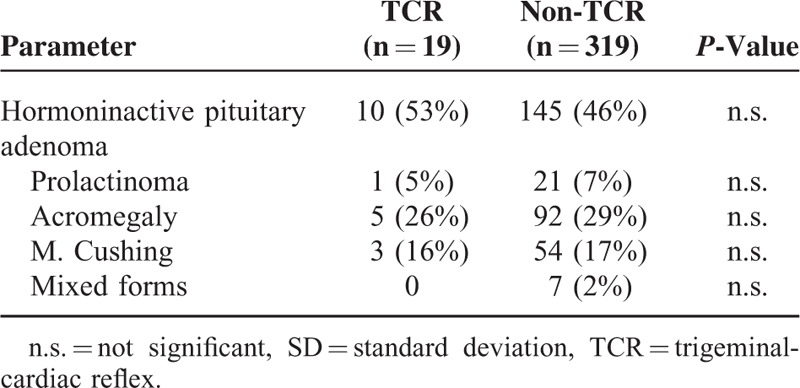
Tumor Histology

**FIGURE 1:**
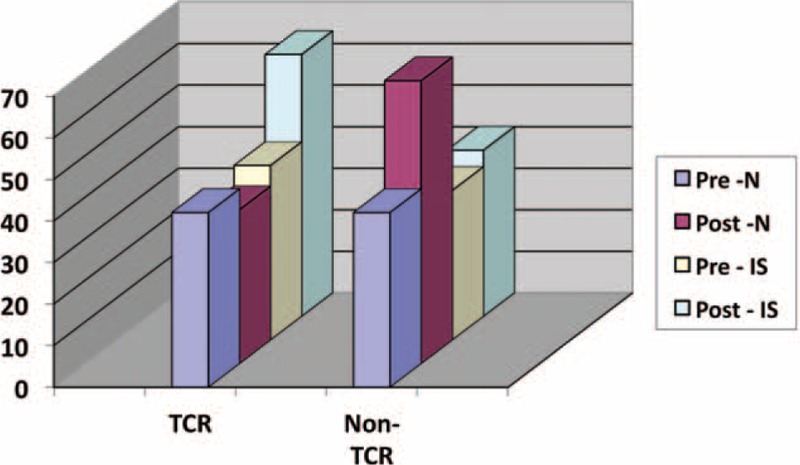
Normalization of pituitary axis in both subgroups. IS = insufficient pituitary axis, N = normal pituitary axis, Post = postoperative, Pre = preoperative, TCR = trigeminal cardiac reflex.

In the TCR group, a preoperatively similar distribution with normal hormonal functions decreased from 42% to 37% after operation (*P* < 0.03) (see Tables 2 and 4). Inversely, the percentage of patients with pituitary insufficiency showed a statistically significant increase after operation (42% to 63%, *P* < 0.03) (see Figure 1). Thus, in comparison to the non-TCR group, patients of the TCR group showed a significant worse outcome regarding normalization of pituitary function (*P* < 0.03) being 3.15 (95% confidence interval, 1.15–8.68) times more likely (see Figure 1). The gender has no influence on this hormonal outcome changes.

**TABLE 4:**
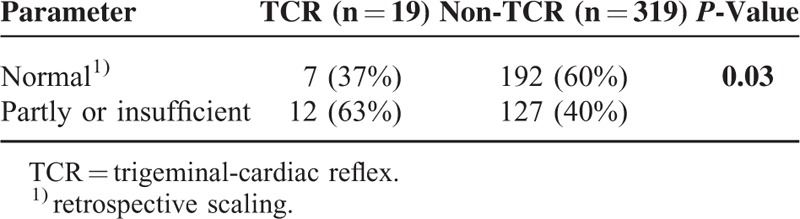
Postoperative Pituitary Hormonal Axis of the 2 Subgroups

## DISCUSSION

In the recent years, the occurrence of a TCR has been observed and documented in different neurosurgical procedures by numerous research groups all over the world,^3,5,8–17,26–61,68–86^ even so the preferred reported region, but rarely outside our group,^28–29,45,49–50^ remains the pituitary region. However, the pathophysiology of TCR is not yet fully elucidated^15–16,50,56,68,70,75,78–79,81,83–95^ and its impact on postoperative outcome remains still subject to further research.

The intraoperative occurrence of the TCR was found to be 6% in the current retrospective study and therefore was significantly lower than in previously published data^3,5,10–11^ where higher prevalences (8%–11%) were advocated. This fact of the current study may be explained by already highly standardized and experienced pituitary operations procedure in our hospital (see for example 41–43) that is known and generally accepted for its world class pituitary surgery. Differences in anesthesia and surgical technique among different institutions along with surgeon's experience and increased awareness could be a possible explanation for these variations.^75^

Intraoperative occurrence of TCR and its relation to postoperative outcome was first observed by Gharabaghi et al in their study on vestibular schwannoma resection.^10,11^ With an overall hearing preservation of 47%, 89% of the patients in the TCR group and 49% of those in the non-TCR group experienced deterioration in hearing function postoperatively.^11^ In this study, larger size of tumors as well as an intraoperative TCR was associated with a significantly worse postoperative hearing function.^11^ These data were further confirmed by Schaller et al in their own follow-up study.^7^ Overall, hypotension following TCR is therefore now regarded to be a negative prognostic factor for hearing preservation in patients undergoing vestibular schwannoma surgery.^7,10,11^

The present retrospective observational study focused on the correlation between intraoperative occurrence of TCR and postoperative (functional) outcome after pituitary surgery. The occurrence of intraoperative TCR and its increased risk with postoperative insufficiency of pituitary hormonal function could be easily plausible if the TCR would occur more frequently in larger tumors due to a consecutive invasiveness into the cavernous sinus or the internal carotid artery, so complete resection of tumor is not possible or an intraoperative damage or compression of the inferior hypophyseal artery or its vessels are more likely; all 3 influencing an impaired blood supply to the pituitary hormone-producing tissue. But there was no significant difference in the tumor diameter between the both subgroups (TCR and non-TCR); however, a tendency to more invasive tumor (Hardy III) in TCR group support this hypothesis at least partly (Table 2), even so the scaling was done retrospectively. Another and more plausible further explanation may be by the TCR occurrence induced intrapituitary ischemia by severe intraoperative bradycardia or asystole by the tumor already structurally changed blood vessels that make them more vulnerable to such damage and that might have negative influence on the postoperative pituitary hormonal functions.^46^ Apart from such possible confounders, the avoidance of TCR will, without any doubt, significantly increase postoperative outcome after pituitary surgery.

A retrospective observational study with risk profile is always difficult because of the often—as also in our study—limited number of patients for subgroup analysis and a therefore consecutive often limited statistical power. Additionally, in such retrospective studies certain variables are not available and/or confounders are present, but not excluded from evaluation. But the previously retrospective gained knowledge in TCR research (see for example^3,5,6,8,12,27^) are now also confirmed by various prospective, partly randomized, studies; also in important anatomical areas for the here nearly exclusively used transsphenoidal approach.^31^ However, prospective cohort studies are considered to be inefficient for such relatively rare outcome as it was the TCR in the present study. All in all the here gained results are the best evidence that we can have, but they are generalizable based on previous comparisons between retrospective and prospective studies on the same topics in TCR. The external validity of the present study is high. First as we could achieve a sufficient statistical power (>80%) and second as we had very uniform process conditions (same, very experienced operator; highly standardized process in a high-volume clinic).

During the last 20 years, there are mentioned numerous risk factors in the literature that may influence or at least predispose the occurrence of a TCR^21,96^ and could therefore be possible confounders for such an analysis as done in this present work. However, as recently examined by Meuwly et al,^96^ much of these previously reported risk factors seems to be more or less anectodical or at least not based on a sufficient scientific basis. However, we set strict inclusion criteria in the present retrospective study as defined earlier by Schaller et al^3^ leading to exclusion of a predominantly part of this potential confounders and we have additionally used a highly standardized protocol that all has spread more or less equally the remaining confounders on both subgroups so that the current results might be directly applicable to the daily clinical practice. This present retrospective study has therefore substantially imposed the importance of the TCR in neurosurgery and particularly in skull base surgery.^96^ According to the current results showing that the TCR also influence the functional hormonal outcome in pituitary surgery, TCR must now be considered not only as an intraoperative phenomenon but also a relevant factor for the postoperative functional outcome, especially in skull base surgery.^7,96^ These new and important findings should therefore be included into daily clinical routine.

## CONCLUSION

This is the first report clearly demonstrating the impact of TCR on the functional hormonal outcome after pituitary surgery. Our results provide strong evidence that the TCR should be considered as a prognostic factor for hormonal outcome in pituitary surgery. It seems that gender has no influence on the TCR. The relatively small number of cases included in this retrospective study restricts its only indicative value and the study should therefore be especially having preliminary character for more extensive studies to examine further details. However, the current findings should yet influence clinical routine.
